# Medical and Physical Intelligence

**Published:** 1804-06-01

**Authors:** 


### MEDICAL AND PHYSICAL INTELLIGENCE.

---

To the Editors of the Medical and Physical Journal.

Gentlemen,

YOU have fallen into a very great error in your critique of my "Researches into the Properties of Spring Water," and I am confident you must wish that the correction of it should be as widely diffused, through the medium of your Journal, as the error itself. You suppose that I attribute the inefficacy of the sulphureous test to detect lead dissolved in common water to the very *small* quantity which it can take up. Thus you say, "The author concludes with giving the chemical experiments which have enabled him, as he he supposes, to detect lead, in *minuter proportions*, than any hitherto known method has been able to do. Again, where the quantity is too *small* to be indicated by the test, &c." But this is entirely to misconceive my meaning, even my words, (See Researches, p. 19) and the object of my experiments. It is not the *smallness* of the quantity, but the *mode of combination* which causes the metal to escape the action of preparations of sulphur, which in the case of this particular solution are no tests at all. In two examples I have shewn, that by a little preparation, the whole body of the water became tinged by sulphurated hydrogen, though this same substance produced no discolouration previous to the preparation. (See p. 165. G). As there is not the smallest reason to think, that the solutions in these specimens were stronger than usual, it is probable, that in all cases there is lead enough to produce an evident discolouration, were it not that the mode of combination prevents the union of the metal and the sulphur. What this mode precisely is, it is not easy fully to demonstrate. A very learned friend conjectures, that the lead is dissolved galvanically. I have offered a different hypothesis, of which I shall be very little tenacious, if a better can be proposed. The weakest possible solutions of common salts of lead, as the acetite, nitrate, &c. are discoloured by sulphurated hydrogen, which is so delicate a test, that it would be ridiculous to ascribe diseases to quantities of this poison, so small as not to be detected by it, on this account only.

I am glad of this opportunity of doing justice to Dr. Withering, by correcting a mistatement of my own. I have said, (p. 32) that no instances are on record of saturnine colic having been excited by these peculiar solutions. But I find in Dr. Withering's "Account of the Foxglove, (case 52) two instances of colics, which he attributed to the water of leaden pumps; besides a third case of an ill state of health, from what had been supposed an irregular gout, ascribed by him to the same cause. Dr. Withering did not at all suspect, that the metal could be dissolved by the water: on the contrary, he expressly says, "Perhaps, in this instance, the metal was so minutely divided by abrasion, as to be mechanically suspended in the water." However, the cases are told in such a manner, as to shew that this eminent physician suspected such water to have deleterious properties; and I am very happy to find my own opinion sanctioned by so grave an authority.

Fourcroy, in the Connoissances Chimiques, (sect. vi. art. 17), has cited some experiments of M. Luzuriaga, to prove that by agitating lead with common water in contact with air, carbonate of lead is formed, which the water dissolves; and adds, that the solution may be discovered by hydrosulphurets. These conclusions are in direct opposition, not to my experiments only, but to those of Bergman, of Baker, and Heberden. But it may be useful to discover the cause of such opposite results; and still more to collect, at some future time, as large a body of evidence as possible sible, on a subject which is obviously of the first interest to society. I shall be obliged, therefore, to any of your correspondents, who will point out where the experiments of M. Luzuriaga are to be found.

WILLIAM LAMBE.

*King's Road, Bedford Row, May 7th,* 1804.

---

### ROYAL JENNERIAN SOCIETY for the EXTERMINATION of the SMALL-POX.

The 'Anniversary Dinner' of this Society was held this day, May 17, 1804, (being the birth-day of Dr. Jenner) at the Crown and Anchor Tavern, in the Strand, at which were present about three hundred members; his Grace the DUKE of BEDFORD, President, in the Chair.

After the healths of their Majesties and of all the branches of the Royal Family, the Patrons of the Institution, which were drank with enthusiasm; that of the immortal discoverer, and of the noble promoters and friends of his discovery, were received with heartfelt ardor.

Mr. Travers, jun. recited, in a very excellent stile, an extract from a poem, written by Mr. R. Bloomfield, called *Good Tidings, or News from the Farm,* which was greatly applauded.

The report brought up by Mr. Travers, sen. and most impressively delivered by him, afforded the highest gratification to the philanthropic and benevolent supporters of the discovery, which forms a new æra in the history of medicine, and which, originating in our isle, already carries consolation to every quarter of the globe.

He gave an account of the great exertions that had been made by the Society, and the very liberal contributions of many noblemen, gentlemen, and ladies. Among other facts, he stated, that by the influence of vaccination, communicated through the medium of the different Societies, the annual deaths from the small-pox had been materially diminished. Through this Society, in co-operation with others, the vaccine system had been propagated in Asia and America. At Constantinople a house had been opened for the purpose of Vaccination; and the Turks, although so much averse to innovation, had embraced the system with the greatest eagerness. In India, the Hindoos, from their religious veneration of the cow, had most materially benefited by this mode of inoculation, and he might almost assert that millions had already been saved by vaccination. In America, the Canadian Indians came down the country many hundred miles to get the matter; and thus whole tribes escaped the effects of that malignant and fatal distemper, the small-pox.

Mr. Travers afterwards stated, that thirty-four persons were now on the establishment of the indigent blind, of whom no less than fourteen owed their blindness to the small pox; and it was to be hoped hoped that by the introduction of vaccination, that institution would ultimately be rendered unnecessary.

The numbers inoculated by the Society in the first year of its establishment were 8333. The persons and places, extending to every quarter of the world, furnished with matter from the central house alone (which has always been done free of expence as well there as at all their stations) amounted nearly to 3000, which had been supplied with 7048 charges or packets. And it is worthy of remark, that in no single instance has the matter furnished by this institution been found to produce a spurious disease. In consequence of this success, medical practitioners in many parts of the United Empire, more especially in the neighbourhood of Portsmouth, in Yorkshire, and the west of Ireland, have lately become particularly desirous of being supplied from thence.

The resident inoculator (Dr. Walker) also made a short report, which gave great satisfaction. He stated the contents of letters he had received from several ladies of distinction and clergymen, who, in their respective neighbourhoods, had successfully introduced the benefits of vaccination by means of virus which he had sent them. Reports were also received from the Jennerian Societies, which correspond with that of the metropolis; and others are rapidly multiplying in different parts of the empire.

### COW-POCK INOCULATION.

On Monday, April 30, ult. there was a most respectable and agreeable Annual Meeting of the Governors of the Original Vaccine Pock Institution, No. 44, Broad Street, Golden Square, established January 1800. Before dinner the Earl of Cholmondeley, the President, being in the chair, the Continuation of the Report was read of the Investigation of the Laws of Agency of the Vaccine-Pock Matter, and of the Practice and Proceedings of the Institution, as written by the Physicians, Drs. Pearson, Nihell, and Nelson.

Among the resolutions were:

1. That the thanks of this meeting be given to Drs. Pearson, Nihell, and Nelson, for their able Report.

2. That this Report be printed under their direction.

3. That the thanks of this Meeting be given to the whole of the Medical Establishment for their gratuitous services.*

Till the Report be printed it may be interesting to our Readers to lay before them an extract relating to the effect of the new inoculation, in diminishing the mortality of the small-pox, concerning which such contrary statements have been published by persons either unacquainted with the facts, or from self interest.

One of the objects of this Institution has been to furnish instruc-

* Not one of the medical officers receive any pecuniary reward; on the contrary, they are all among the most liberal subscribers, from themselves and from their friends, as appears by the printed list.

tions for the practice of Vaccination; and this has been done by shewing patients to visitors and students, and by public lectures, as well as by written and printed papers. This establishment has accordingly disseminated the new inoculation through many parts of the world. By this time its instructions and matter have introduced the Vaccine Inoculation in New South Wales, as it did before in Paris, Vienna, &c. &c.

It may be expected from the extensive practice of vaccination, (this institution alone having vaccinated and been the immediate means of vaccinating 60,000 persons) that the fatality of the small-pox must have been diminished. That diminution however does not appear, for the Bill of Mortality for London reports 1202 to have died in the year 1803, whereas 1111 died in the year 1799, 522 only in 1797, and 1040 in 1795; and although the number has been greater in the intermediate years, yet, still the number last year, viz. 1202 is not much less than the mean number for each year during any five years for half a century past.

How it has happened that no diminution of mortality is yet perceived may easily be understood, when it is considered that the number inoculated for the cow-pock are chiefly those who would have been inoculated for the small-pox, and that therefore the same proportion remained for the natural small-pox. Hence, hitherto, vaccine inoculation, like the small-pox inoculation, is only a benefit to individuals; but this benefit is very much greater than the variolous inoculation, although the variolous inoculation, by preventing the natural small-pox, was, till the vaccine inoculation, the greatest benefit in Physic.

Prejudice, indolence, ignorance, want of opportunity, still occasion inoculation of either kind to be but partially adopted in society at large. How far laws might be established, or means be found out, for every person within a certain period after birth being inoculated, cannot be discussed on this occasion, however important this question may be for the legislature.

After dinner, the President being obliged to attend the House of Peers, the following statement was delivered by Lord Petre, one of the Vice Presidents.

The grand object of this Institution on its establishment, a little more than four years ago, was, to extinguish the small-pox by substituting for it the inoculation of the cow-pock; but however great the obligations of the public were to Dr. Jenner, the promulgator of the leading practical fact in 1798; to Dr. Pearson also in 1798, and to Dr. Woodville in 1799, for their investigations to justify the new inoculation, still a professed Institution was wanting, in order to,

First, Extend by gratuitous inoculation the history of the vaccine pock, of which, comparatively, but little was still known.

Secondly, To diffuse the knowledge of the new practice.

Thirdly, To preserve a succession of patients for matter for the use of the public.

To

To what extent the first of these designs, may be judged from the report published in a former year, and from the papers distributed containing directions for inoculation, and will be judged of further by the report this day read and ordered to be printed.

With regard to the second part of this plan, the diffusing the knowledge of the new inoculation, the practice has been publicly carried on twice a week ever since January 1800, at which a great number of practitioners and many students have been present for instruction.

Institutions, confessedly upon a similar plan, have been established in other places, and instructions for the practice have been disseminated in every part of the world.

With regard to the third part of the plan, the succession and the supply of matter; as might be supposed, the numbers inoculated during the years 1800 and 1801, were not considerable. However, a register has been kept, and more or fewer cases have been registered twice a week from January, 1800, up to the present time; thereby affording a body of evidence of very nearly 2000 patients, which have been subjects of observation during this space of time. Such a long and uninterrupted course of observation, we apprehend, has no where else been pursued; the advantages of observation of even half a dozen patients a week for 200 to 220 weeks successively, over any greater than the total number here inoculated, but in a few months or weeks, can well be conceived by those who have ever been employed in observation, and need not be explained.

It appears that not less than 12,000 parcels of matter have been furnished by this institution, and thereby it is estimated fairly, that not fewer than 60,000 persons have been vaccinated with matter directly from this institution, besides an incalculable number from those so vaccinated.

The whole pecuniary expence for these benefits does not amount to much more than three hundred pounds per annum; and although the subscriptions are voluntary, and mostly of small annual sums, and although the institution has sustained great expences and pecuniary losses, chiefly from unfortunately parting with money on a loan, and from being obliged to change their house from the practice, yet there is a surplus of five hundred and fifty pounds stock in the funds, and a respectable balance in the hands of the banker; what is surprising is, that many have received from this institution a reward to submit to the test of small-pox inoculation, and others have been relieved who were in distressed circumstances; hence it is concluded, that there is not to be found an example of even nearly so much benefit to individuals and society at large, at so small an expence. However, although it be very true that, provided the present subscribers be permanent, the income will be adequate to the present expenditure, it is not to be dissembled that the practice and enquiry might be conducted upon a larger scale, and more agreeably to the different officers, if their income would allow it. Accordingly, although it is not the plan of this meeting to to canvass for subscriptions, it is hoped that its friends will thereby be augmented in such a manner as is thought proper, the public having already had an earnest that their benefactions will be wisely employed by the present managers.

---

On Monday, June 4th, a Course of Lectures on Physic and Chemistry will recommence at the Laboratory, Whitcomb-street, Leicester-square, at the usual morning hours, viz. The *Therapeuticks* at a quarter before eight; the *Practice of Physic* at half after eight, and the *Chemistry* at a quarter after nine, by GEORGE PEARSON, M. D. F. R. S. Senior Physician to St. George's-Hospital; of the College of Physicians, &c. A register is kept of Dr. Pearson's patients in St. George's-Hospital, and an account is given of them at a Clinical Lecture every Saturday morning at nine o'clock. The Practice of Vaccination will be shown, and Lectures given as usual, during the summer, at the Institution, No. 44, Broad-street.

---

Mr. SAUNDERS, demonstrator of practical anatomy at St. Thomas's Hospital, purposes to deliver during the summer, a Course of Anatomical Lectures.—The object which he has in contemplation, is to give a compendious view of anatomy, adapted to the practical purposes of medicine and surgery. The parts of the human body which are interesting merely as objects of curious speculation, will not be introduced into these lectures; but the attention will be confined exclusively to those parts that being particularly subject to disease and accident require the application of art, and consequently demand the serious consideration of every medical practitioner and surgeon. The course will comprehend the anatomy of the vital organs, organs of sense, joints, extremities, and all those parts of the human body which are most liable to become the subjects of operative surgery. The demonstrations will be conducted in such a manner as is best calculated to lead to practical inferences, and will be followed by an explanation of the different surgical operations, and, as far as his means permit, of the morbid anatomy of the parts requiring those operations. The Course will consist of thirty lectures, which will be delivered at his house, No. 24, Ely Place, Holborn, and will commence the 14th of June, at eight o'clock in the morning, and be continued every day at the same hour. The terms of attendance two guineas.

---

Dr. HARRINGTON has now in the press, to be ready in June or July, a Complete Overthrow of the French Theory of Chemistry; proving its unstable principles, upon the most clear and satisfactory evidence.

---

END OF VOL. XI.

